# Meta-analysis of active tuberculosis gene expression ascertains host directed drug targets

**DOI:** 10.3389/fcimb.2022.1010771

**Published:** 2022-10-05

**Authors:** Nirmaladevi Ponnusamy, Mohanapriya Arumugam

**Affiliations:** Department of Biotechnology, School of Biosciences and Technology, Vellore Institute of Technology, Vellore, Tamil Nadu, India

**Keywords:** tuberculosis, meta-analysis, gene ontology, pathway enrichment, genetic variants, drug repurposing

## Abstract

Multi-drug resistant tuberculosis still remains a major public health crisis globally. With the emergence of newer active tuberculosis disease, the requirement of prolonged treatment time and adherence to therapy till its completion necessitates the search of newer therapeutics, targeting human host factors. The current work utilized statistical meta-analysis of human gene transcriptomes of active pulmonary tuberculosis disease obtained from six public datasets. The meta-analysis resulted in the identification of 2038 significantly differentially expressed genes (DEGs) in the active tuberculosis disease. The gene ontology (GO) analysis revealed that these genes were major contributors in immune responses. The pathway enrichment analyses identified from various human canonical pathways are related to other infectious diseases. In addition, the comparison of the DEGs with the tuberculosis genome wide association study (GWAS) datasets revealed the presence of few genetic variants in their proximity. The analysis of protein interaction networks (human and *Mycobacterium tuberculosis*) and host directed drug-target interaction network led to new candidate drug targets for drug repurposing studies. The current work sheds light on host genes and pathways enriched in active tuberculosis disease and suggest potential drug repurposing targets for host-directed therapies.

## Introduction

Tuberculosis (TB) is an infectious disease which remained throughout human history. *Mycobacterium tuberculosis* (*Mtb*) is the main causative agent of TB. Around 10% of individuals develop TB when exposed to *Mtb* and 5% of the infected individuals develop TB within 1-2 years while the remaining 5% develop the disease at any other time (Frieden et al., 2003). Active TB has higher burden of TB when compared to latent TB (Lee, 2016). Individuals with compromised immune systems, such as people with HIV, diabetes or people with constant tobacco use are at high risk of falling ill.

TB is the 13^th^ leading cause of death globally in 2020. Around 86% of new cases reported around the world in 2020 were contributed majorly by China, Indonesia, the Philippines, Pakistan, Nigeria, Bangladesh and South Africa with India leading the list (WHO, 2021). Over the period of time *Mtb* has adopted newer subversion strategies to successfully evade the host immune system enabling it to reside in the host resulting in latent or active disease manifestation (Behar et al., 2010; Ernst, 2018).

The infection results in a complex dynamics between the host and pathogen triggering various immune signalling cascades and cross-talks between molecular components (Casadevall and Pirofski, 2000). Several contributing factors associated with the disease were identified through genetic and biochemical experimental studies. The recent surge of omics data has further aided in understanding of factors influencing predisposition of the disease and markers associated with the disease severity.

With increased availability of gene expression data, studies based on TB blood transcriptomics offers a robust approach to study the immunology of TB. The comparative studied of healthy and TB cohorts shed light on differentially expressed genes (DEGs) and also allow observations of such DEG upon vaccine/drug treatment. Further the DEG analysis also aids in understanding of regulatory mechanisms contributing to the functional consequences.

In the current study, a statistical meta-analysis was carried out using whole blood expression profiles from infected TB patients to identify key human transcriptomics signatures characteristic of the disease. The study also utilized host genetic disease association and drug-repurposing analyses to further to prioritize the results. In addition, the gene ontology and pathway-based annotations identified genes and pathways significantly altered in the diseased condition.

## Materials and methods

### Dataset selection and processing

The whole-blood microarray gene expression profiles of patients with active pulmonary tuberculosis and healthy cohorts were retrieved from NCBI GEO (Barrett et al., 2013). The datasets were further filtered based on the following conditions: (i) The expression profiles were from human patients affected by tuberculosis undergoing no prior treatment, (ii) Only samples from active tuberculosis patients and control groups were considered, (iii) The datasets should include both healthy controls and patient group, (iv) The patient or the control group should not be infected with any other secondary diseases, (v) The patient and control group should include more than 5 samples each.

The background corrected files were processed using limma package in R (Ritchie et al., 2015). The data were quantile normalized, log transformed and missing values were removed. The probe identifiers were converted to Entrez gene IDs. If multiple probes are mapped to a single gene, then the average expression value of the probes were used for the gene. Post normalization, individual datasets were subjected to Principal Component Analysis (PCA). PCA was carried out to observe a distinct separation between the active and control samples.

### Statistical meta-analysis and validation

Statistical meta-analysis was carried out using NetworkAnalyst (Zhou et al., 2019). NetworkAnalyst interprets gene expression data including meta-analysis, tissue specific PPI networks, gene regulatory networks, gene co-expression networks along with networks for toxicogenomics and pharmacogenomics studies. The pre-processed expression values were used as input for the web tool. Differential expression analysis for each dataset was performed using limma with false discovery rate (FDR) cutoff of 0.05. The batch effects were adjusted using ComBat method. The corrected datasets were merged and statistical meta-analysis was carried out using INMEX. The combined effect size method for meta-analysis was used to generate the results. The random effect model which encloses cross-study heterogeneity was used for meta-analysis. Differentially expressed genes (DEG) were obtained using FDR cutoff of 0.01 in the meta-analysis. The DEGs with absolute combined effect size > 1.5 were chosen for genetic variant analyses and drug interaction analyses.

### Validation of meta-analysis

The strength of the results obtained from meta-analysis was further validated by comparing the genes expressed in latent and control samples from the same datasets. Partial Least Square Discriminant Analysis (PLS-DA) was applied to the DEGs. Significant model was selected by 7-fold cross validation. The model performance was evaluated using the area under the Receiver Operating Characteristic (ROC) curve (AUC). All the above validation process was carried out using mixOmics package in R (Rohart et al., 2017).

### Gene and pathway enrichment

Gene and pathway enrichment analysis was carried out using DAVID web server to identify significantly enriched Gene ontology (GO) biological processes (BPs) and KEGG pathways, which were ranked based on the hypergeometric test with FDR cutoff of 0.05 (Sherman et al., 2022). DAVID web server offers functional annotation and enrichment analyses of gene lists provided by the user.

### Protein-protein interaction network construction

A comprehensive human protein-protein interaction network (hPPI) was constructed. High confidence, experimentally verified interactions extracted from STRING database was used for the construction (Szklarczyk et al., 2021). The STRING database integrates known and predicted associations between proteins encompassing physical interactions and functional associations. Similarly, the pathogen proteins interacting with host DEG were mined from various literature sources (Rapanoel et al., 2013; Penn et al., 2018; Augenstreich and Briken, 2020; Verma et al., 2022). These data were used to construct human protein – *Mtb* protein interaction network (hmPPI).

The highly interconnected components of the hPPI and hmPPI were identified using the Cytoscape plugin CytoHubba. CytoHubba is a user-friendly interface to explore important nodes in biological networks using various topological metrics. The hub genes of the networks were identified using the topological metrics degree and Maximal Clique Centrality (MCC) (Chin et al., 2014).

### Drug-target interaction

The drug compounds interacting with DEG were retrieved from DrugBank Version 5 (Wishart et al., 2018). DrugBank is a comprehensive database which holds information about drugs, their mechanisms, interactions and targets. Drugs with experimental or clinical evidence for direct interactions with the protein were selected. Drugs with pharmacological actions as the same direction of the DEG and drugs with unknown pharmacological actions were excluded. Only DEGs with a combined effect size greater than 1.5 were considered for the analysis.

### Genetic variant analysis

The genetic differences between tuberculosis-affected and healthy individuals can give a mechanistic insight about the disease and functional implication of the affected gene. The single nucleotide polymorphisms (SNPs) proximal to the DEG were obtained from GRASP database (P-value < 5e-8) (Leslie et al., 2014). GRASP database encloses deeply extracted and annotated database of genome-wide association studies (GWAS) results enclosing more than 6.2 million SNP-phenotype association. Similarly, the regulatory SNP were retrieved from Slidebase database using the enhancer regions of the DEG (Ienasescu et al., 2016). SlideBase offers a new way of selecting genes, promoters, enhancers and microRNAs that are preferentially expressed/used in a specified set of cells/tissues. The genomic regions in linkage disequilibrium (LD) with the SNP were collected from SNAP (Johnson et al., 2008). The association between a gene and its corresponding SNP was prioritized based on the overlap between the genomic location of the DEG or its enhancer and LD region of a SNP. The query tool SNAP enables the identification of single-nucleotide polymorphisms (SNPs) and annotate nearby SNPs in linkage disequilibrium (proxies) based on HapMap project results.

## Results

**Figure 1:**
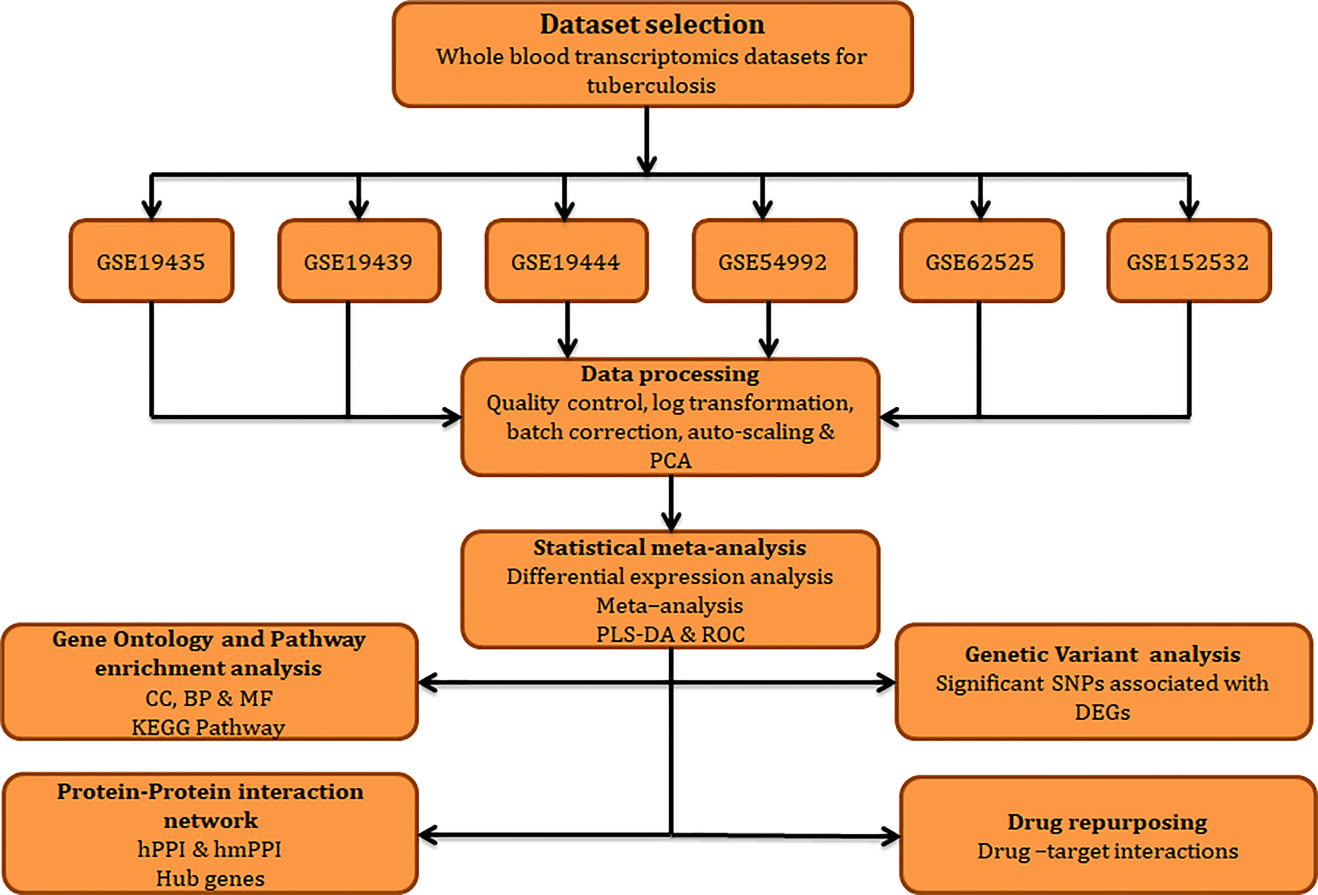
Workflow adopted in this study.

The work plan and approaches implemented in the current study is illustrated in **Figure 1**.

### Identification and validation of DEG from meta-analysis

The database querying and filtering identified around 149 GEO microarray datasets for TB-related host response at the time of study (June 2022). Further, filtering based on the study inclusion criteria, a total of six datasets enclosing control and active TB samples were selected for next set analyses (**Table 1**).

**Table 1:** List of GEO datasets used in the meta-analysis.

Dataset	PMID	Platform	Samples*				DEGs
			Active	Latent		Control
GSE19435	20725040	Illumina	7	0		12	2793
GSE19439	20725040	Illumina	13	17		12	1600
GSE19444	20725040	Illumina	21	21		12	2063
GSE54992	24647646	Affymetrix	9	6		6	4454
GSE62525	26818387	Phalanx	7	7		7	8739
GSE152532	34555657	Illumina	17	69		11	586

*Only untreated samples were considered for the analysis.

**Figure 2:**
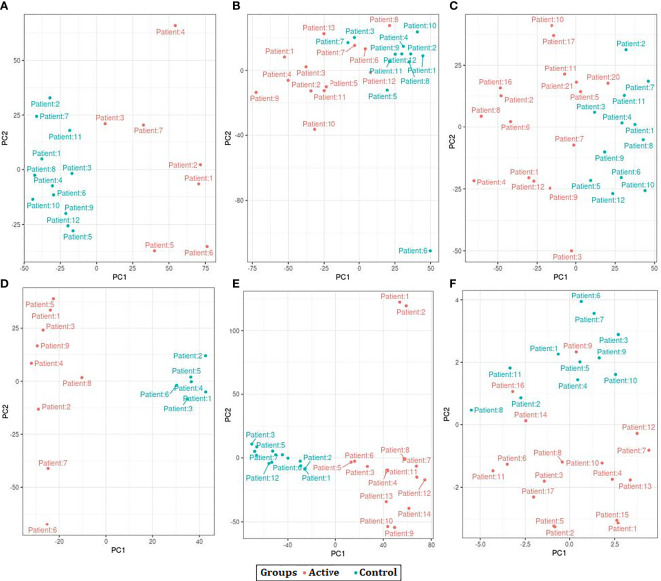
Principal Component Analysis (PCA) plots showing the separation control and active samples across all datasets used in the study. **(A)** GSE19435 **(B)** GSE19439 **(C)** GSE19444 **(D)** GSE54992 **(E)** GSE62525 **(F)** GSE152532.

The results of PCA indicated that the samples were clustered based on the observations of the study. However, after batch correction the samples were clustered based on the disease condition as active TB and control. We also observed a few samples outside the clusters before and after the batch correction procedure (**Figure 2**).

**Figure 3:**
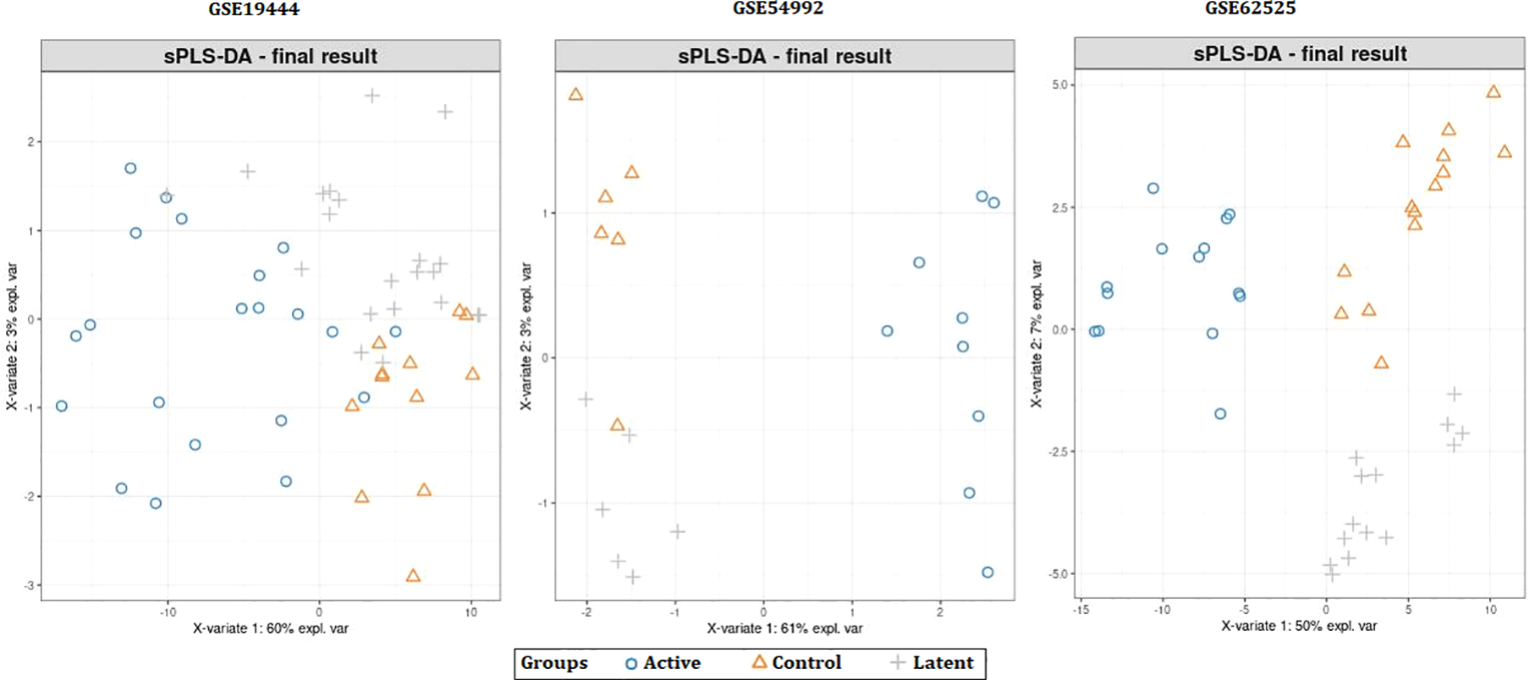
Partial least squares-discriminate analysis (PLS-DA) plots showing differentiation between the control and TB samples.

**Figure 4:**
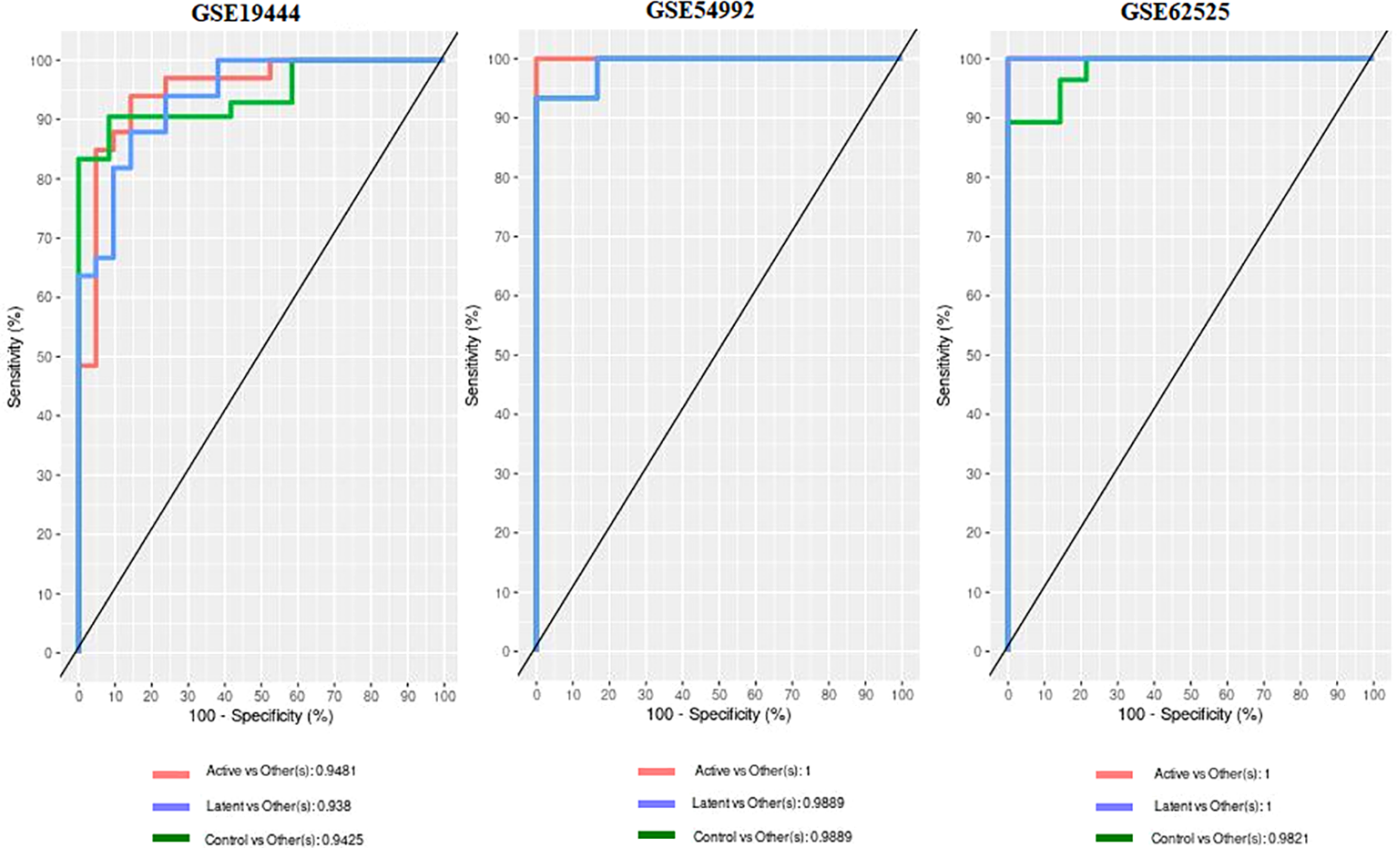
Receiver operating characteristic (ROC) and Area under curve (AUC) from the PLS-DA on the 113 DEGs data.

When the datasets DEG were compared, we identified genes regulated in the same direction. The meta-analysis identified a total of 2038 DEG of which 861 genes were up-regulated and 1177 genes were down-regulated (**S. Table 1**). Further analysis identified a total of 113 genes (up-regulated – 24 and down-regulated- 89) with absolute combined effect size as a reference for the log2 fold change (logFC) greater than 1.5 (**Table 2**). S1PR1 ranks first among the up-regulated genes. S1PR1 expression is associated with lymphocyte recirculation. Similarly, FCGR1B is the top-ranking gene which is down-regulated in the active TB. To assess the results obtained from the meta-analysis, we validated the 113 genes with logFC > 1.5 in three datasets GSE19444 (Illumina), GSE54992 (Affimetrix) and GSE62525 (Phalanx) from different platforms. The PLS-DA models showed good sensitivity (above 85%) and specificity (above 83%) in all three datasets. The control, active and latent TB samples formed three different clusters marking clear differentiation (**Figure 3**). The ROC plot for the models suggest that the PLS-DA model can distinguish active tuberculosis samples from both latent and control groups with high true positive and low false positive rate (**Figure 4**). These measures show that DEG can act as biomarkers for the detection of active TB cases.

**Table 2:** List of top 20 DEGs (absolute combined effect size > 1.5) identified in the meta-analysis.

DEG	Fold change in meta-analysis	FDR P-value in meta-analysis
S1PR1	2.0215	0.000123
ZNF91	1.8479	0
PASK	1.7283	3.29E-06
PIK3IP1	1.7086	7.08E-09
CCR7	1.699	2.22E-06
GRAP	1.6851	1.70E-07
PDCD4	1.6847	0.01366
LAX1	1.68	0.005286
SLC38A1	1.6476	1.59E-06
ABCB1	1.6387	0.001482
FCGR1B	-2.2365	0
VAMP5	-2.2051	6.28E-12
GBP5	-2.0828	0
LY96	-2.0495	3.19E-10
TNFSF13B	-2.0145	3.61E-06
PSMB9	-1.9828	6.79E-13
CASP1	-1.9688	0
IL15	-1.9614	0
RNF135	-1.9367	0.002385
BATF2	-1.9255	0

### Identification of significantly enriched gene ontologies

The functional GO was carried out for the up and down regulated DEG identified by the meta-analysis (**Table 3**). The GO analysis identified that up-regulated DEG significantly involved in cellular components (CCs) were nucleoplasm, nucleus and cytosol. For GO BP analysis, the DEG showed involvement in mRNA splicing, *via* spliceosome, cytoplasmic translation and rRNA processing. Similarly for GO molecular function (MF) analyses, the DEGs were majorly enriched in RNA binding, protein binding and ATP binding. The GO CC analysis of down-regulated DEGs showed involvement in extracellular exosome, cytosol and lysosome. The GO BP analysis identified involvement in defense response to virus, innate immune response and response to virus. The GO MF analysis showed enriched functions such as protein binding, protease binding and MHC class I protein binding.

**Table 3:** Top 10 significantly enriched Gene ontologies.

Regulation	Cellular component (cc)	Biological Process (BP)	Molecular Function (MF)
Up	GO:0005654-nucleoplasm	GO:0000398-mRNA splicing, via spliceosome	GO:0003723-RNA binding
	GO:0005634-nucleus	GO:0002181-cytoplasmic translation	GO:0005515-protein binding
	GO:0005829-cytosol	GO:0006364-rRNA processing	GO:0005524-ATP binding
	GO:0005737-cytoplasm	GO:0006357-regulation of transcription from RNA polymerase II promoter	GO:0003724-RNA helicase activity
	GO:0016020-membrane	GO:0006281-DNA repair	GO:0016887-ATPase activity
	GO:0005730-nucleolus	GO:0006412-translation	GO:0003676-nucleic acid binding
	GO:1990904-ribonucleoprotein complex	GO:0006355-regulation of transcription, DNA-templated	GO:0003677-DNA binding
	GO:0022626-cytosolic ribosome	GO:0000122-negative regulation of transcription from RNA polymerase II promoter	GO:0003735-structural constituent of ribosome
	GO:0016607-nuclear speck	GO:0006397-mRNA processing	GO:0004386-helicase activity
	GO:0005840-ribosome	GO:0006338-chromatin remodelling	GO:0003682-chromatin binding
Down	GO:0070062-extracellular exosome	GO:0051607-defense response to virus	GO:0005515-protein binding
	GO:0005829-cytosol	GO:0045087-innate immune response	GO:0042802-identical protein binding
	GO:0005764-lysosome	GO:0009615-response to virus	GO:0003725-double-stranded RNA binding
	GO:0005765-lysosomal membrane	GO:0045071-negative regulation of viral genome replication	GO:0004298-threonine-type endopeptidase activity
	GO:1904813-ficolin-1-rich granule lumen	GO:0032731-positive regulation of interleukin-1 beta production	GO:0001730-2'-5'-oligoadenylate synthetase activity
	GO:0016020-membrane	GO:0006954-inflammatory response	GO:0042803-protein homodimerization activity
	GO:0035580-specific granule lumen	GO:0032755-positive regulation of interleukin-6 production	GO:0002020-protease binding
	GO:0010008-endosome membrane	GO:0050729-positive regulation of inflammatory response	GO:0061133-endopeptidase activator activity
	GO:0005886-plasma membrane	GO:0006915-apoptotic process	GO:0050786-RAGE receptor binding
	GO:0035578-azurophil granule lumen	GO:0032757-positive regulation of interleukin-8 production	GO:0004175-endopeptidase activity

### Identification of significantly enriched pathways

The pathway enrichment analysis implemented using DAVID identified various dysregulated pathways mediated by the DEGs (**Table 4**). The up-regulated DEGs showed enrichment of pathways involved in Spliceosome, Ribosome and Nucleocytoplasmic transport. We also observed pathways overlapping with other infectious diseases such as Herpes simplex virus 1 infection and Coronavirus disease - COVID-19.

**Table 4:** List of top 10 significantly enriched human pathways in the meta-analysis.

Up regulated pathways	Down regulated pathways
hsa03040:Spliceosome	hsa04621:NOD-like receptor signaling pathway
hsa03010:Ribosome	hsa04142:Lysosome
hsa03013:Nucleocytoplasmic transport	hsa05164:Influenza A
hsa05168:Herpes simplex virus 1 infection	hsa05132:Salmonella infection
hsa04660:T cell receptor signaling pathway	hsa05160:Hepatitis C
hsa03018:RNA degradation	hsa04145:Phagosome
hsa05340:Primary immunodeficiency	hsa05169:Epstein-Barr virus infection
hsa03008:Ribosome biogenesis in eukaryotes	hsa05152:Tuberculosis
hsa05171:Coronavirus disease - COVID-19	hsa05162:Measles
hsa05166:Human T-cell leukemia virus 1 infection	hsa05140:Leishmaniasis

The down-regulated DEGs showed involvement in NOD-like receptor signaling pathway, Lysosome and other infectious disease pathways such as Influenza A, *Salmonella* infection, Hepatitis C and Epstein-Barr virus infection. We also observed the presence of Tuberculosis pathway in the list. The presence of tuberculosis pathway in our analysis indicated that the genes identified in the meta-analysis demonstrate their significant association with the disease.

### Protein–protein interaction network construction

**Figure 5:**
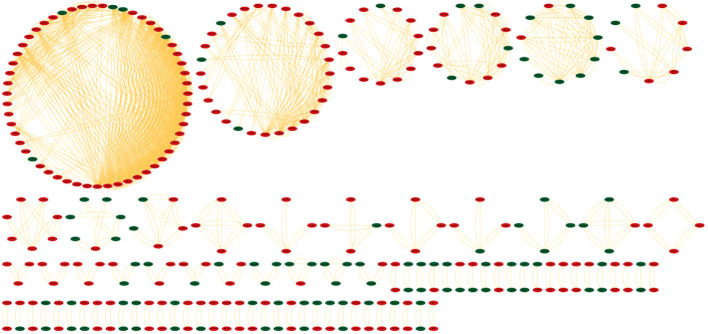
Protein-protein interaction network (hPPI) of the 2038 DEGs in the meta-analysis. The up-regulated genes are colored in red and down-regulated genes are colored green. The edges are represented as orange lines.

Identifying the physical interactions between the proteins will provide clues to combat infection. The mapping of 2038 DEGs along with their partners resulted in a network enclosing 325 nodes and 1460 edges (**Figure 5**). The average number of neighbours in the hPPI was 15. Around 323 genes showed direct interactions with their partners. The top ten hub genes which showed overlaps in degree and MCC measures are RPL10A, RPS4X, RPS16, RPS23, RPS3, RPS13, RPL7A, RPL4, RPS5 and RPS6. All the identified hub genes were ribosomal proteins involved in RNA binding.

**Figure 6:**
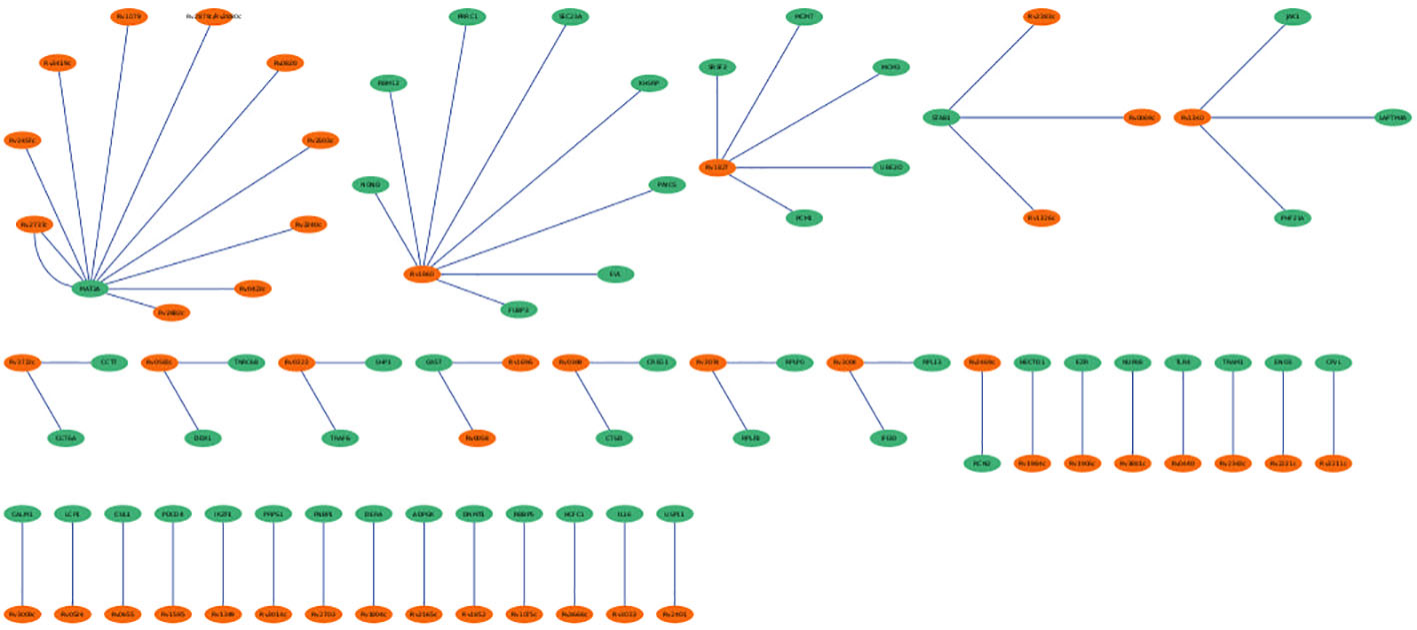
*Mtb*-host protein-protein interaction network (hmPPI). The *Mtb* proteins are colored green and human proteins are colored in orange. The edges are represented as blue lines.

The hmPPI interaction network enclosed 99 nodes and established 66 connections with an average of 1 connection between the neighbours (**Figure 6**). Due to the availability of limited *Mtb*-host protein-protein interactions we did not observe any hub genes based on the topological metrics.

### Drug – target interaction

The DEGs from the study were queried against DrugBank to mine drugs targeting genes which may be used for repurposing against TB. A total of 22 drugs targeting 8 DEGs were obtained after the screening process (**Table 5**). Among them each compound showed association with at least one target gene with few exceptions such as ABCB1 which showed interaction with 14 drugs. Human immunoglobulin G (DB00028) acting on C5 and FCGR1B were also observed. FYN kinase targeted by the Fostamatinib was one among the up-regulated genes.

**Table 5:** List of drugs in DrugBank targeting DEGs in the meta-analysis.

DEG	Fold change	Drug name	Pharmacological action	Disease treated	Mechanism of action
TSPO	-1.5792	Chlormezanone (DB01178)	Agonist	Muscle spasms	Inhibition of the ascending reticular activating system; Blocking the cortical and limbic – reticular pathways.
		Zopiclone (DB01198)	Agonist	Insomnia	Inhibitory actions of GABA.
FYN	1.5683	Fostamatinib* (DB12010)	Inhibitor	Immune thrombocytopenia	LRRK2 and spleen tyrosine kinase inhibition.
		Dasatinib* (DB01254)	Multitarget	Chronic myeloid leukemia	Src family tyrosine kinase inhibitor.
C5	-1.5903	Human immunoglobulin G (DB00028)	Binder	Immunodeficiency; Autoimmune disorders	Prevent infection by attaching to the surface of invading pathogens and aiding in their disposal before they can infect cells.
		Eculizumab (DB01257)	Antibody	Autoimmune disorders	Inhibition of complement complex C5b-9.
TNFSF13B	-2.0145	Belimumab (DB08879)	Neutralizer	Systemic lupus erythematosus; Active lupus nephritis	Blocks its interaction with B cell receptors - transmembrane activator and calcium-modulator.
ABCB1	1.6387	Medroxyprogesterone acetate	Inhibitor	Secondary amenorrhea; Renal carcinomas	Production of gonadotropin inhibition.
		Fentanyl (DB00813)	Inhibitor	Anesthesia	Inhibition of nerve activity.
		Voacamine (DB04877)	Inhibitor	Multidrug-resistance in tumor cells	It is possibly a substrate for P-glycoprotein (P-gp), an efflux pump responsible for multidrug resistance in tumor cells.
		Tocofersolan (DB11635)	Antagonist	Vitamin E deficiencies	It acts as a free radical chain breaking molecule, halting the peroxidation of polyunsaturated fatty acids and maintaining both the stability and integrity of cell membranes.
		Hycanthone (DB14061)	Inhibitor	Schistosomiasis	
		Concanamycin A (DB14062)	Inhibitor	Fungal infection	Binds to specific cell-surface receptors.
		Dexverapamil* (DB14063)	Inhibitor	Cardiac arrhythmias	Anti-arrhythmia drugs are divided into four main groups: calcium channel blockers; beta-adrenergic blockers; sodium channel blockers; and repolarization prolongers.
		Emopamil* (DB14064)	Inhibitor	Renal injury	
		Lomerizine (DB14065)	Inhibitor	Migraines	Inhibition of calcium influx through cellular membranes.
		Tetrandrine* (DB14066)	Inhibitor	Immunosuppression; Proliferation	Inhibition of calcium influx through cellular membranes.
		Dofequidar (DB14067)	Inhibitor	Neoplasm	Inhibits or prevents the proliferation of NEOPLASM.
		Dexniguldipine (DB14068)	Inhibitor	Hypertension	It exhibited a binding affinity for P-glycoprotein, assuming it could impede P-glycoprotein pumping and modify multidrug resistance.
		Desmethylsertraline (DB14071)	Inhibitor	Depressive disorder	
		Reversin 121 (DB14072)	Inhibitor		
FCGR1B	-2.2365	Human immunoglobulin G* (DB00028)	Antagonist	Immunodeficiency; Autoimmune disorders.	Blocks gamma Fc receptors, preventing the binding and ingestion of phagocytes and suppressing platelet depletion.
MYC	1.5067	Aspirin* (DB00945)	Down regulator	Inflammation; Migraines; Cardiovascular events	Blocks prostaglandin synthesis; With high dose for COX-2 inhibition.
		Nadroparin* (DB08813)	Inhibitor	Prophylaxis of thrombotic events	It inhibits coagulation cascade.
S1PR1	2.0215	Fingolimod* (DB08868)	Modulator	Multiple sclerosis	To reduced lymphocyte circulation into the central nervous system.

*used for tuberculosis via varying mechanism.

### Genetic variant analysis

The detection of drug targets which has human genetic support by its involvement in the disease pathology may aid in success of the treatment against the disease by preventing late stage clinical failures. The evidence of involvement in the disease by the 2038 DEGs was retrieved from genome-wide association study (GWAS) datasets. A total of 483 TB-related SNPs were obtained from GRASP database. An overlap between the LD region with a SNP and the DEG location or the enhancer region suggest strong association between the SNP and that particular gene. A total of 33 genes showed association with TB-related SNPs (**Table 6**).

**Table 6:** List of DEGs proximal to TB-associated SNPs.

DEG	SNP associated with the gene
ABCB1	rs1128503, rs1045642
ACSS1	rs6138553
ACTA2	rs1800682
BLK	rs2254546
CCR7	rs11659024
CD5	rs10897125
CD6	rs10897125
CIRBP	rs2285899
COX19	rs11761941
CSTA	rs10934559
ENTPD1	rs10882657
FAS	rs1800682
FBXO31	rs10779243
GBP2	rs12121223
GBP5	rs2146340
GMFG	rs10412931
HLA-DPA1	rs3129750
KIF1B	rs11121555
LAP3	rs10939733
MDC1	rs1317834
MICB	rs2532929
OAS1	rs10774671
PBX4	rs1859287
PGD	rs11121555
PSMB10	rs12102971
PSMB8	rs3129750
PSMB9	rs3129750
SCO2	rs12148
SP110	rs3948464
TAP1	rs3129750
TAP2	rs3129750
TIMM10	rs2649662
WDR6	rs1134591

## Discussion

### Meta-analysis of active tuberculosis samples

The meta-analysis of transcriptomes in this study identified S1PR1 as an up-regulated gene with highest fold change. S1PR1, by the detection of its ligand S1P in the blood and lymph, is crucial for naive lymphocytes to access the circulatory system. S1P-S1PR1 signaling is crucial for regulating immune cell development and function. S1P-S1PR1 signaling is needed for mature thymocytes to leave the thymus and for T/B cells to leave secondary lymphoid organs and enter the blood or lymph in both homeostatic and pathological situations (Sinha et al., 2009; Zachariah and Cyster, 2010; Allende et al., 2010; Zhang et al., 2012). S1PR1 analog therapy raises IL-6 and lowers IL-10, but it can’t stop the mycobacterial infection inside the cell (Arish and Naz, 2022). Consequently, the diminished expression of S1PR1 causes retention of naïve T cells in lymphoid tissues (Skon et al., 2013). Similarly, FCGR1B is the top ranking gene with highest fold change and is down-regulated in the active TB condition. The FCGR1B gene is a member of immunoglobulin G, which binds directly with pathogens and neutralizes them. Changes in the Fc gamma receptors affect the response of a host to infection (Song et al., 2017). The FCGR1B gene aids in the host’s immune response during a mycobacterial infection. According to Maertzdorf’s research, people with TB and LTBI had more DEGs than uninfected individuals (Maertzdorf et al., 2011). In a different study, Satproedprai et al. found that the overexpression of FCGR1B in response to bacterial infection caused a humoral immune response and contributed to the development of lung inflammation (Satproedprai et al., 2015). Our results showed downregulation of FCGR1B, probably as a result of active TB.

The enrichment of spliceosomes and lysosomes in GO, indicates its crucial role in active TB infection. The functions of spliceosomes are also regulated differently in infected macrophages. The lysosomes protect against *Mtb* by controlling how *Mtb* moves through the lysosomes and stopping it from spreading in cells. Pre-mRNA splicing plays a crucial role in regulating gene expression and protein diversity. The Serine/Arginine rich (SR) proteins are the major components contributing to the selective splicing mechanism. The disruption in the RNA splicing mechanism can lead to crosstalk in the intricate network interactions. The *Mtb* infection alters the patterns of alternate splicing within the macrophages by affecting the expression of SR proteins (Zhang et al., 2018).

The evolution of *Mtb* infection has proven its ability to successfully gain access to host cellular components needed for its survival before the initiation of innate antimicrobial response. The process is accomplished by altering various immune response elements such as interferon (IFN)-induced transmembrane (IFITM) gene family members. The IFITM members receive signals for their activation through type I and II IFN stimulation to preclude the establishment of productive infection. Ranjbar et al. show that IFITM proteins inhibit *Mtb* intracellular growth, indicating that they may contribute to host defense against intracellular bacterial infection (Ranjbar et al., 2015). The IFITMs act on host membrane fluidity at the sites of viral entry by preventing the formation of viral fusion pore. In addition, they increase the trafficking of trapped viruses to the lysosome for its degradation. However, *Mtb* alters this phagocytic mechanism by switching off the acidification of the phagosomes mediated by the IFTIM family members. One such example is the vacuolar ATPase, a mediator of endosomal acidification which is excluded from *Mtb*-containing phagosome by *Mtb*’s bacterial tyrosine phosphatase (Ranjbar et al., 2015).

In addition, pathways related to immune mediated cascades such as T cell receptor signaling pathway, Th17 cell differentiation and NF-kappa B signaling pathway were also observed (Urdahl et al., 2011). The order of appearance of T cell receptor signaling pathway down the list indicate that the onset of genes contributing to this pathways are delayed. Nuclear factor-kappa B (NFκB) pathway mediates pro-inflammatory responses which are required by the host to control of many microbial pathogens. The activation of NFκB has proven to increase the viability of intracellular *Mtb* in human macrophages by preventing apoptosis and autophagy (Bai et al., 2013). The lysosomal and pathogenic pathways indicate the dominance of these pathways used by the pathogens to avoid lysosomal targeting. They function by actively manipulating the host vesicular trafficking and reside in a vacuoles altered from the default lysosomal trafficking (Sachdeva and Sundaramurthy, 2020). The overlap of infectious disease pathways signals the usage of similar players for evading the infection and using host counterparts to reproduce.

The PPI networks reveal the involvement of major players contributing to the infection. The hPPI network identified ten hub genes which play crucial role in the infection. All these identified hub genes were ribosomal proteins involved in RNA binding. These RNA-binding proteins play critical roles in co- and post-translational regulation. Due to the distinct differences in ribosome structure between *Mtb* and the host, the ribosome is a multiprotein complex, and the protein-protein interactions of its subunits may be an appealing target for novel antibiotics (Lin et al., 2012). Earlier report based on microarray expression analysis such as Wang et al., also reported up-regulation of 22 unique ribosomal proteins in tuberculosis infection (Wang et al., 2003). In the current work we identified the involvement of 10 (RPL10A, RPS4X, RPS16, RPS23, RPS3, RPS13, RPL7A, RPL4, RPS5 and RPS6) unique ribosomal proteins in the active disease stage with high degree of connectedness. However, the significance of these genes is unclear and requires future studies. The hmPPI network showed MAT2A of the host protein interacted with ten *Mtb* proteins. MAT2A catalyse the conversion of L-methionine to S-adenosyl-L-methionine in cysteine and methionine metabolism. The *Mtb* protein partners also perform similar function, for example MetK which is a methionine adenosyltransferanse (Wang et al., 2003).

The meta-analysis identified the involvement of various kinases in active TB. The drug bank list also narrowed down few drugs acting on kinases. Various classes of tyrosine kinase inhibitors exhibit distinct mechanism of action to inhibit phagocytosis of tubercle bacilli in dose and time-dependent manner. Early studies have proven that tyrosine kinase inhibitors including Dasatinib, Bosutinib, Imatinib, Nilotinib, Ponatinib, Nintedanib, Fostamatinib and Tirbanibulin reduce the growth of intracellular *Mtb*. The ligation of complement receptors by *Mtb* plays a major role in stimulation of tyrosine phosphorylation (Schlesinger and DesJardin, 2022). Focusing on drugs targeting these proteins can act as a starting point for the development of host mediated drug repurposing studies. The genetic-variant analysis recognized genes contributing to drug resistance such as ABCB1 (Pontual et al., 2017) and susceptibility to latent tuberculosis such as SP110 and OAS1 (Chang et al., 2018; Leisching et al., 2019). Analyzing the genetic variants of the tuberculosis patients before starting any treatment regimens is highly suggested to prevent late stage failures.

## Conclusion

The current study focuses on meta-analysis and highlights host genes and pathways crucial for tuberculosis disease. The DEGs identified in the current work shed light on promising drug targets for host-directed repurposing therapies. The work also suggests considering the genetic variants associated with the TB-related genes to enhance the success rate of therapies in individuals affected with tuberculosis. Future studies assessing the behavior of the identified DEGs during and after the treatment can ascertain their involvement in the disease pathogenesis and progression.

## Data availability statement

The datasets presented in this study can be found in online repositories. The names of the repository/repositories and accession number(s) can be found in the article/**Supplementary Material**.

## Author contributions

Collected data, Implemented the analysis and Manuscript writing: NP. Conceived and designed the analysis: MA. All authors contributed to the article and approved the submitted version.

## Funding

This research did not receive any specific grant from funding agencies in the public, commercial, or not-for-profit sectors.

## Acknowledgments

We would like to thank Vellore Institute of Technology (VIT) for providing computational facility.

## Conflict of interest

The authors declare that the research was conducted in the absence of any commercial or financial relationships that could be construed as a potential conflict of interest.

## Publisher’s note

All claims expressed in this article are solely those of the authors and do not necessarily represent those of their affiliated organizations, or those of the publisher, the editors and the reviewers. Any product that may be evaluated in this article, or claim that may be made by its manufacturer, is not guaranteed or endorsed by the publisher.

## Supplementary material

Supplementary Table 1 List of 2038 DEGs identified by meta-analysis.
